# Online high-resolution real-time monitoring techniques for anions in river water

**DOI:** 10.1007/s10661-025-14954-y

**Published:** 2026-01-13

**Authors:** Julia Arndt, Anna-Lena Gerloff, Alex Zavarsky, Michael P. Schlüsener, Arne Wick, Lars Duester

**Affiliations:** 1 Federal Institute of Hydrology, Am Mainzer Tor 1, 56068 Koblenz, Germany; 2 North Rhine Westphalia Office for Nature, Environment and Climate, Duisburg, Germany

**Keywords:** Ion chromatography, Colorimetry, UV–Vis sensor, Nutrients, Rhine

## Abstract

High-resolution real-time monitoring gains increasing importance in environmental monitoring. We employed and evaluated three real-time monitoring techniques to monitor anions at the river Rhine in Koblenz, Germany. These are a sensor for nitrate in situ and ex situ, a colorimetric device for nitrate and nitrite, and an online ion chromatography (IC) for fluoride, bromide, chloride, nitrate, nitrite, phosphate, and sulfate. The optical sensor performed well ex situ while in situ, a higher maintenance effort was needed due to fouling processes. Colorimetry failed to analyze and notify when solutions fouled and required a very high amount of maintenance to reliably analyze nitrate and nitrite. The online–IC was feasible for analyzing seven anions with a low amount of maintenance once the required infrastructure for the instrument was provided. The online high-resolution IC data was compared with conventional monitoring data with, e.g., a routine interval of two weeks, which was shown to fail to capture peak concentrations during high discharge events. Additionally, to monitor short-term events, like the shortest registered peaks of 5 h, monitoring intervals of at least 2 h at the river Rhine are needed. By extending the IC time series to 2.5 years supported by principal component analysis, a dendrogram clustering, and event analysis by QC-diagrams, two major groups of anions were visible. The first group includes discharge-driven anions such as chloride, bromide, sulfate, and fluoride, with fluoride being additionally influenced by the equilibrium precipitation of fluorite. The second group consists of seasonal-driven anions like nitrate, nitrite, and phosphate, with phosphate being also affected by short-term events and hence, remains to be an important indicator in online monitoring setups. To get a complete picture, anion analyses can be combined with, e.g., element or non-target analyses.

**Supplementary Information:**

The online version contains supplementary material available at 10.1007/s10661-025-14954-y.

## Introduction

The term “real-time monitoring” is frequently used in environmental monitoring without having a precise definition. It emphasizes that single processes are different than usual: the faster availability of data or the higher monitoring frequency (or a combination of both aspects). Terms used alike are “in-time monitoring” or “online monitoring” (Capodaglio et al., 2016), with the latter being even more common. While online monitoring also describes the location of sampling and analysis, which is in a bypass of the main stream, the terms real-time and in-time only refer to the time in which analysis is conducted and data are available. Thus, the location of analysis can also be directly in the main stream (Arndt et al. (2022) and references therein). Automated processes from “industry 4.0” have been applied for a long time, yet are just now being transferred to environmental monitoring. As an example, big data collection and analysis are applied by big search engines, online booking, and shopping platforms for a long time and now also emerge in environmental monitoring (Najah Ahmed et al., 2019).

Anions reveal the status of the ecosystem regarding nutrients like nitrate, nitrite, and phosphate and potentially induce eutrophication when present in excess. The main source for nitrate in surface waters is agriculture, land use in general (Liang et al., 2020; Neill, 1989), where it is applied as fertilizer and subsequently is washed out by precipitation and transferred to ground and surface water (Bijay & Craswell, 2021). An additional source for nitrate is sewage (Xu et al., 2021). Nitrite occurs when nitrate is transferred under anaerobic conditions in the presence of a carbon source by microbes (Fanning, 2000), and it is more toxic (ATSDR, 2017). In contrast, phosphate has its main source for surface water presence from wastewater treatment plants (WWTP). In modern plants, phosphate is removed from wastewater either by biologic elimination or by chemical precipitation, so only lower concentrations enter the rivers (80% of EUs’ WWTP, Pistocchi et al., 2023). The retained phosphor can eventually be recovered for reuse in a (nowadays still challenging) process (Di Capua et al., 2022). In surface waters, the nutrients phosphate and nitrate, combined with higher temperatures and enough sunlight (e.g., in spring or during summer), can induce algae blooms of cyanobacteria (Yang et al., 2008). These blooms consume the abovementioned nutrients, cause oxygen deficiency at night, and may release cyanotoxins into the water (Ernst et al., 2005), which may harm the ecosystem, but also can be harmful to humans (Kubickova et al., 2019). Therefore, it is of utmost importance to monitor these nutrients, even more in high waste water treatment infrastructure environments.

The current state of the art for anion analyses used in routine (offline) freshwater monitoring involves manual sampling and subsequent analysis by ion chromatography (IC, EN ISO 10304–1 (EN, 2009a)), as long as water does not exceed a specific salinity depending on the chromatographic system in use (Gros, 2013). Some anions are also analyzed by wet-chemical techniques like colorimetry, e.g., phosphate by ISO 15923–1 (ISO, 2013), nitrite (NEMI, 1995), and nitrate (NEMI, 2005), by continuous flow analysis (CFA, Cheong et al. (2024)) or electrophoresis (Oehrle, 1996). Optical methods without any addition of chemicals (Pellerin et al., 2013) work for optically active substances like nitrate by absorption of a specific wavelength, e.g., Rieger et al. (2008) or Boënne et al. (2014).

For real-time monitoring of nitrate, nitrite, and phosphate in wastewater treatment plants, optical sensors or colorimetry are frequently used (Glasgow et al., 2004; Ingildsen and Olsson, 2002; Korostynska et al., 2012; Kruse, 2018; Lu et al., 2023; Tran et al., 2022). Being optically active anions, nitrate and nitrite can be detected by UV–Vis sensors at the wavelengths of 220 and 210 nm (Singh et al., 2019). In general, optical sensors are deployed for real-time monitoring and have the advantage of immediate data availability (Thakur and Devi 2024) and references therein. The advantage of sensors often is their response time, their low maintenance, and low infrastructural needs, as they fundamentally only demand electricity. The sensors can be applied to analyze inline or online (in the main stream or a bypass of the main stream). Optical sensors are highly selective and have high precision (Pellerin et al., 2016). A disadvantage of sensors is their sensitivity to the characteristics of turbidity or colored water (e.g., during flooding or induced by humic substances) at each site (Kumar et al., 2024).

Colorimetric devices which analyze nitrate, nitrite, and phosphate by addition of complexing agents are commercially available and need some infrastructure, which is at minimum a stock supply of the respective complexing agent, but can also involve deionized water, a chemical waste, and further chemical agents. For phosphorus, in situ analyses have been done based on the molybdate blue method (Dadi et al., 2023).

An IC can analyze all listed anions above in one analytical run. It is routinely applied for laboratory analyses. Automation of sample introduction into an IC, in most laboratory applications, only reaches as far as an autosampler being attached and prepared samples and solutions being provided. For real-time monitoring, the instrument and its surrounding infrastructure have to be set up close to the monitored river and an automated water sample preparation by filtration is needed. This can be regarded as a disadvantage as it poses a lot of effort, but has been done for several rivers in recent years as mentioned below. In technical process analysis, real-time analysis by IC is applied frequently. This is also shown by the availability of ready-to-use process assemblies by the larger IC manufacturers. In process monitoring, most infrastructure demands are already available onsite, e.g., housing, energy supply, air conditioning, or tap water supply. As an example, online-ICs are employed in process analytics in semiconductor industries (Ehmann et al., 2006) or used in process wastewater monitoring for cationic amines coupled to a MS/MS (Wortberg & Kurz, 2019). In real-time monitoring of surface water, a self-made 3D-printed IC has been applied for 7 days to monitor nitrate and nitrite (Murray et al., 2020). A river laboratory has been set up for anions and cations analyzing every 40 min by Floury et al. (2017) or every 30 min for 28 days by von Freyberg et al. (2017). In the latter two studies, river water sampling was automated by a pump in the river, following automated filtration (to 0.2 µm or 1 µm, respectively) before the sample was transferred automatically to the IC. The same technique as in Floury et al. (2017) has been applied in three different watersheds using a sampling frequency of 7 h (Wang et al., 2024) and for another watershed with a sampling frequency of 1 h (Brekenfeld et al., 2024).

To support river basin managers and those who monitor water quality, we compare high-frequency real-time monitoring by online-IC with colorimetry for the anions nitrite and nitrate and the optical UV–Vis sensor for nitrate. Our primary focus was the instruments’ ease of manual handling concerning 24/7 monitoring and the quality of monitoring data. Additionally, the application of the UV–Vis sensor for monitoring of nitrate and dissolved organic carbon (DOC) is compared in situ and ex situ (directly inside the river and in a bypass of the river in the monitoring station). Next to this, to complement the picture, we also present 2.5 years of high-frequency monitoring data from the river Rhine by automated online-IC with a resolution of 40 samples per day in order to provide a better understanding of the benefits of high-frequency monitoring. With these two parts, this study compares time series of the anions analyzed, evaluates them for similarities and patterns over time and between the anion concentrations, and gives an economic and analytic summary of the instrument compared to deliver advice to those who run or manage monitoring stations or networks.

## Materials and methods

### Location

**Fig. 1:**
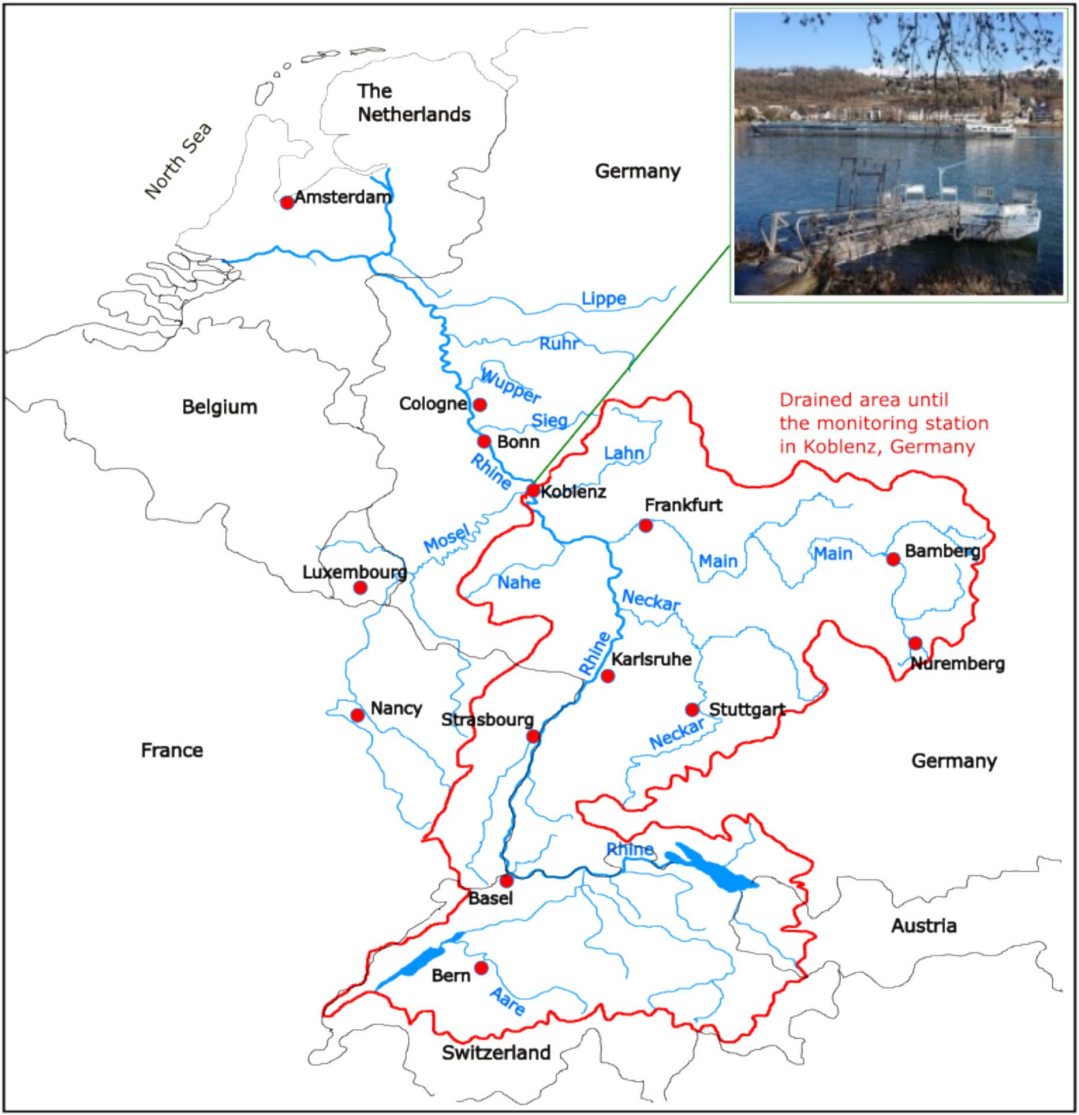
Geographic location of the monitoring site (river km 590) including the river Rhine, its tributaries, and the drained area until the monitoring station in Koblenz. The river Moselle joins the Rhine approximately 0.5 km downstream of the monitoring station in Koblenz, while the river Lahn joins the Rhine a few hundred meters upstream on the right bank side. The monitoring station is on the left bank side of the river Rhine. The river Lahn contributes to the discharge of the Rhine measured at the monitoring station, but not to the chemistry, as it is not considered to be fully mixed during these few hundred meters. The small picture shows the pontoon used for in situ analyses. It also contains the immersed pump which supplies the monitoring station for ex situ analyses. Figure adapted from the International Commission of the Protection of the Rhine (ICPR, 2018)

The Rhine River, one of Europe’s most important waterways, flows through six countries, including Switzerland, France, Germany, and the Netherlands, where it reaches the North Sea. At the monitoring station Koblenz (Germany, Rhine km 591), it already drained an area of 110,000 km^2^, together with its major tributaries Neckar, Main, Nahe, and Lahn (Fig. 1). The river is used by the economy and industry, e.g., for traffic, cooling water, ecosystem services as drinking water production (especially downstream of Koblenz by bank filtrates), and as a recreational area. These usages can interfere and pose multiple conflicts of interests. The Rhine water monitoring station in Koblenz is located on the left bank side. Here, the tributary Lahn only merges into the river Rhine on the right bank side a few hundred meters upstream and thus cannot be considered to have been fully mixed with the river Rhine. All the other tributaries upstream can be considered to have been fully mixed with the river Rhine due to the large distance and several bends and are consequently analyzed at the monitoring station on the left bank side.

### Monitoring setup

The monitoring station is situated about 200 m from the Rhine. In the monitoring station, a second pump is used (AxFlow, Germany) to permanently supply an aliquot to two consecutive filters. The water is pre-filtered first with a 3D-printed nylon mesh with 1 cm long oval holes (Ultimaker S5, The Netherlands), and then a subsequent at 0.2 µm filtration is performed using a cross-flow filter system (4H–Jena, Germany with filters from MiniKros, Repligen, The Netherlands). This creates about 30–150 mL of filtrate per minute, which then is led to a flow-through cell to supply the analytical instruments.

### Implementation of the optical sensors

Two identical optical UV–Vis sensors equipped with an optical analysis distance of 1 cm were used (OPUS sensor, TriOS, Germany) for the analysis of the parameters nitrate, dissolved organic material (equivalent to DOC = DOC_eq_), spectral absorption coefficient at 254 nm (SAK_254_), and total suspended solids (TSS_eq_). One was placed in situ at the pontoon (Fig. 1), while the other one monitored ex situ in a sensor basin of the monitoring station (May – June 2020). Sensors were factory-calibrated, and calibration was checked using a nitrate standard serial dilution of three concentrations (5, 10, 15 mg∙L^−1^-NO_3_) prepared from a 1000 mg∙L^−1^-NO_3_ stock solution of p.a. grade (VMR, US). For DOC_eq_ as a sum parameter, measurements were calibrated using ex situ samples and laboratory analyses by DIC-DOC analyzer (DIMATOC 2100, DIMATEC Analysetechnik GmbH, Germany). Data sets with high turbidity were indicated incorrect by the sensor and thus neglected as turbidity interferes with optical measurements at all wavelengths. During the time of the comparison experiment, both sensors were manually cleaned after every manual sampling. The sensors were set to one optical analysis every 10 min, with prior automated cleaning of the measurement windows by an automated wiper. Turbidity was analyzed additionally by infrared measurement of scattered light (Solitax, HACH; ISO-70227–1, (EN., 2019)). The error estimation for the UV–Vis sensors given by the manufacturer is 5% of the total value + 0.1 mg mg∙L^−1^-NO_3._ The same is valid for DOC_eq_.

### Implementation of the online-IC

A laboratory Ion Chromatography (IC; 940 Professional IC Vario, Metrohm), equipped with a standard analytical column for anions (Metrosep A Supp 5–250 + Guard, Metrohm), an Eluent Production Module, a Liquid Handling Module, and a 947 UV detector (Metrohm), was coupled to an ultrapure water system (18.2 MΩ, PurelabFlex, ELGA/Veolia, Germany). Samples are taken from the flow-through cell that was constantly flushed with fresh filtered river water. The IC analyzes approximately one sample every 30 min and automatically checks its own calibration every 10 samples by two quality control standards. This frequency allows on average to analyze 40 samples per day. If a quality control is not within an 85–115% recovery rate, a re-calibration is done automatically. After that, quality controls are analyzed again, and if met, the next ten samples are measured. If quality control standards do not reach a recovery between 85 and 115% after a re-calibration, 100 samples are analyzed automatically in order to avoid a gap of analyses until it is possible to interfere manually by the user, e.g., by providing fresh stock solutions. Thus, it is guaranteed to provide consecutive river data which can be corrected afterward and to avoid a loop of quality controls caused by external parameters. The online sequence of the IC is illustrated in Fig. SI 1. Briefly, the results of the online IC were cross-checked with grab samples and laboratory analyses at the beginning of the monitoring period. Focusing on the aim to make the online IC run on its own, a cross-checking technique with quality control standards was developed. These are the aforementioned quality control checks every ten samples to assure data quality. The IC analyzes seven parameters: fluoride, bromide, chloride, nitrate, nitrite, sulfate, and phosphate. Calibration ranges can be found in Table SI 2. For calibration preparation, standards of p.a. grade were used (1000 mg∙L^−1^ standards of each element, VMR). The calibration was adjusted to these ranges based on the monthly long-term data of the ICPR monitoring program https://iksr.bafg.de/iksr/.

### Implementation of colorimetry

Colorimetric devices for nitrate, nitrite, and phosphate (Systea Analytica Technologies, Italy) required filtered river water and therefore were also supplied with 0.2 µm filtered water in the monitoring station. Additionally, several chemical solutions were required for analysis using the vanadium reduction method for nitrate (NEMI 9171 (NEMI, 2005)) and for nitrite likewise (SM 4500-NO2-B (NEMI, 1995)). Required solutions for colorimetric analysis were prepared according to the producers’ recommendations. All chemicals used were of p.a. grade. They were freshly prepared every 4–6 weeks and kept at 4 °C in the dark, if not needed. Quality control standards were measured manually at the same device (as offline samples) every 14 days to assess the device performance, quality of method, and quality of solutions. River water was monitored every 3 h. An acceptable error of manual quality controls was ± 15%. If the quality control was not met, the method was checked, the instrument was rinsed with blank solutions, and quality controls were repeated. In case of great differences, solutions were prepared freshly and exchanged.

### Laboratory analysis

For anions’ offline analyses, a second Metrohm IC 940 was used with the same method described above. For the analyses of DOC_eq_, a thermic analytic oxidation was performed by DIC DOC analyzer (DIMATOC 2100, DIMATEC Analysetechnik GmbH, Germany). For colorimetry offline cross-checks of nitrate, cuvette tests (Hach, Germany) were applied.

#### IC

After the automated quality control measurements, data was manually verified (regarding outliers and plausible concentrations). Every sample was corrected using the recovery rates of quality control standards that were analyzed before and after the respective sample. To process the monitoring data and to merge and compare them with the other datasets, IC timestamps were rounded to every 30 min or hourly (i.e., 10:27 was rounded to 10:30).

### Sensors

After first manual checks, sensor data processing was automated using R, filtering for single outliers and outliers due to high turbidity and measurement of air. A running median of about 5 measurements was applied due to the very high analysis frequency of 10 min. Single outliers were only eliminated in cases where (a) the sensor itself showed a potential error for optical measurements and/or (b) the value was back to “normal” in the next measurement.

### Colorimetry

Data from the colorimetric device were (after additional data sorting due to non-standard output format) checked for outliers manually.

### Time series

Device cross comparison was undertaken for the analytes and instruments displayed in Table 1. Similarity of temporal element patterns was identified and supported using cluster analysis applying the average clustering method and a distance calculation by correlation.

**Table 1:** Comparison of instruments and parameters in this study

	Devices for comparison						
Analyte	Online-IC	Offline-IC	Online colorimetry	Offline colorimetry	Sensor in situ	Sensor ex situ	Offline DIC DOC analyzer
Nitrate		X		X	X	X	
DOC_eq_					X	X	X
TSS_eq_					X	X	
Nitrate and nitrite	X		X			X	

Additionally, a principal component analysis (PCA) was applied for the data from IC of the entire monitoring period, including all anions as well as the parameters “discharge” and “seasonality.” To be able to compare anion patterns instead of absolute concentrations in the PCA, concentration time series of every element was normalized using the highest concentration occurring for every element. The seasonality was assessed by introducing a parameter called “day of the year” (DoY) which simply counts upwards every consecutive day and from day 182, which is half the year, downwards again. The data was processed using R version 4.3.3 and RStudio (version 2023.09.1) with the R packages “dplyr” and “dygraph,” and figures were plotted using the R package “ggplot2.”

Up-to-date non-validated high-frequency monitoring data of anions by the online-IC of the last 30 days are available for the monitoring station “Koblenz/Rhine” at https://undine.bafg.de/rhein/guetemessstellen/rhein_mst_koblenz_rh.html.

## Results and discussion

During the study, several methods were compared: (A) The UV–Vis sensor for nitrate and DOC was evaluated for its performance in situ and ex situ. This was done by comparing two identical sensors at these locations simultaneously and by comparing their results with manual samples and offline laboratory analyses conducted by IC, colorimetry, and the DIC–DOC analyzer. (B) The performance of 3 high-resolution online analytical techniques was compared: IC, colorimetry, and the sensor. (C) The time series of all anions analyzed by online–IC were evaluated for patterns, and concentrations were set in relation to discharge. (D) The monitoring intervals were evaluated for the smallest occurring peaks.

### In situ and ex situ comparison with UV–Vis sensor

**Fig. 2:**
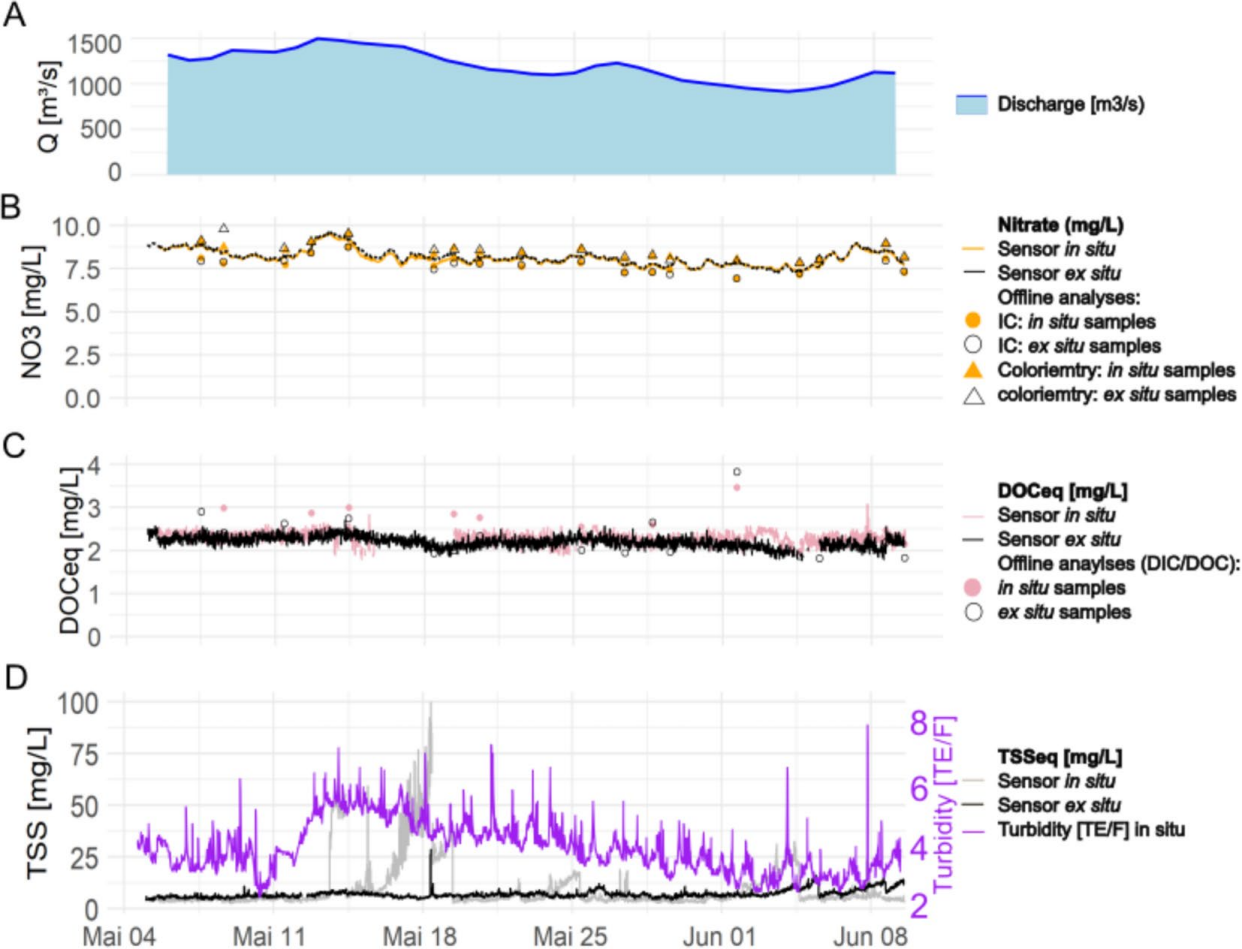
Comparison of in situ and ex situ measurements by UV–Vis sensor. Ex situ and in situ measurements of nitrate, DOC_eq_, and TSS_eq_ are shown as black and colored lines, respectively. Additionally, discontinuous analysis with offline techniques (IC and colorimetry for nitrate and DIC; DOC analyzes for DOC_eq_) were performed (dots and triangles). Turbidity was measured by UV–Vis (= TSS_eq_) in situ and ex situ, and by infrared measurement of scattered light in situ only (TE/F equivalent to FNU). A normalized version is available in the SI (Fig. SI10)

In situ and ex situ analyses were conducted using two identical optical UV–Vis sensors to measure nitrate (NO_3_^−^), as well as the two sum parameters of dissolved organic carbon (DOC_eq_) and total suspended solids (TSS_eq_). The in situ measurements were taken at the pontoon in the river (inline), while the ex situ measurements were conducted in a flow-through basin inside the monitoring station using unfiltered river water (online). Both sensors provided continuous data every 10 min. Additionally, discontinuously manually sampling for offline analyses was performed to assure accuracy of the sensor results. Analyses for nitrate were conducted offline using ion chromatography (IC) and colorimetry, while analyses for DOCeq were performed using a DIC-DOC analyzer (see “Materials and methods” section for details). The results were compared, and the standard deviations of several analyses are presented in this section for comparison (Fig. 2). The respective data of this section are provided in the data repository (Appendix 1 zenodo.org/records/15736566.).

### Nitrate

The nitrate sensor results in situ and ex situ are very similar during the 6 weeks of comparison. The mean difference between the two was 0.04 ± 0.03 mg∙L-1-NO_3_ throughout the whole monitoring time and across all analyses conducted during the 6 weeks (Fig. 2B). This difference is, with a mean concentration of 8.1 mg∙L-1-NO_3_, < 0.5% of the full value and well below the error specification of 5% + 0.1 mg∙L-1-NO_3_ by the manufacturer. Due to nitrate’s full solubility in water and close sensing locations, these close-to-equal concentrations can be explained.

In comparison to the offline analyses by IC and colorimetry, the differences were 0.4 ± 0.16 mg∙L-1-NO_3_ and 0.4 ± 0.17 mg∙L-1-NO_3_, respectively. However, the deviations occurred in the opposite direction, with concentrations being colorimetry > sensor > IC (Fig. 2B). Mean nitrate concentration was 8.1 mg∙L-1-NO_3_ for NO_3_, meaning the relative error between the techniques was < 10%. This error between the different measurement methods is smaller than the error from the sensor, the IC, or colorimetry.

The DOC_eq_ concentrations show a correlation between in situ and ex situ measurements, but the absolute differences are slightly larger than for nitrate, 0.08 ± 0.14 mg·L^−1^, resulting in a higher relative error due to the lower values in the overall results (5.4 ± 4.3%, Fig. 2C). If the optically interfering turbidity measured by the sensor (Fig. 2D) increases, the correlation between the two sensors deteriorates or even becomes unmeasurable. High turbidity has a greater impact on the measurements of DOC_eq_ compared to nitrate. The reason could be that nitrate has a more distinct spectral response with a peak at around 180 nm. In contrast, DOC_eq_ as a sum parameter relies on superimposed features, which are determined by the experience of the manufacturer or the user. However, an additional effect is also present in the UV–Vis data. The fouling effect on the measurement windows (e.g., by algae growth or other biofouling effects) led to biased results, especially for DOC_eq_. This effect can only be partly mitigated by including a cleaner on the sensor (every 10 min). Additionally, it was generally observed that the effect was more pronounced in situ than ex situ. This might also be the reason for errors in the DOC_eq_ detection in situ after the higher discharge. Compared to offline analyses, the DOC_eq_ data differed 1.14 ± 0.55 mg·L^−1^. The difference between offline analyses and the sensor’s data of the respective location (in situ and ex situ) was pronouncedly larger for in situ data of the sensor (Fig. 2). This seems logical due to the calibration of the sensor, which was done using ex situ samples that were analyzed offline (compare “Materials and methods” section).

### TSSeq

Comparing turbidity measured by UV–Vis sensor (Fig. 2D) to the EN ISO (EN, 2019b) method by infrared measurement of scattered light (TE/F equivalent to FNU), the overall trend of infrared measurements can also be noticed in UV–Vis measurements. The higher turbidity is caused by the higher discharge (Fig. 2A).

The comparability between TSS_eq_ data at the two locations is limited as soon as a certain threshold is reached in situ (Fig. 2D). The measurement in the station is clearly smoothed compared to the in situ data, neglecting spike concentrations and accumulation. Most likely, the smoothening is caused by particle fractionation by the inlet and the pump supplying the monitoring station, while the accumulation effect is most likely caused by fouling effects on the sensors.

In conclusion, the UV–Vis analysis of DOC_eq_ is more sensitive to disturbances by fouling and the turbidity than the analysis of nitrate. We also conclude that the monitoring location (in situ vs. ex situ) does not matter for substances that are dissolved in water (excluding oxygen), such as nitrate. However, for particle-driven and particle-impacted analyses, such as DOC_eq_ and TSS_eq_, the monitoring location has an effect on the monitoring results.

### Comparison of the three real-time high-resolution techniques (IC, sensor, and colorimetry) for nitrate and nitrite

From November 2021 to March 2022, all three applied analytical methods for nitrate and nitrite were running in parallel: online–IC, the UV–Vis sensor (ex situ), and online–colorimetry. These techniques analyzed at time intervals of every 30 min (IC), every 10 min (sensor), and every 2–6 h (colorimetry). In this section, we compare the results of nitrate and nitrite obtained from IC and colorimetry, as well as the nitrate results obtained from the sensor. The full data set of this section is provided in the data repository (Appendix 2 zenodo.org/records/15736566.).

**Fig. 3:**
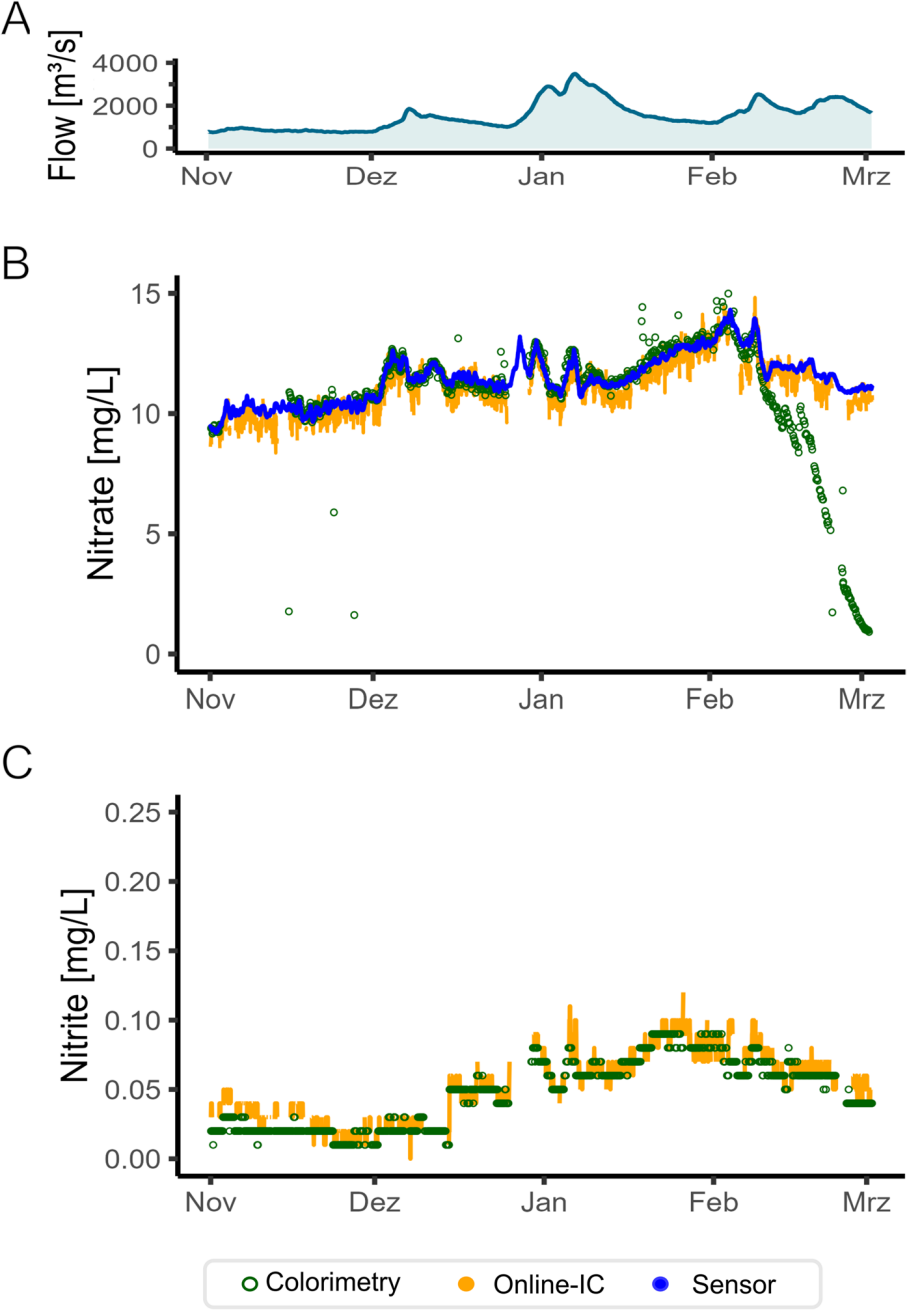
Parallel monitoring from November 2021 to March 2022 using the three online monitoring techniques: Colorimetry (green dots, every 2–6 h), IC (orange dots, hourly means of 30 min measurements), and sensor (blue dots, hourly means of every 10 min measurements) for the two parameters: **A** nitrate and **B** nitrite in mg∙L^−1^-NO_2_

In the period of investigation, nitrate concentration ranged from 8 to 15 mg∙L^−1^-NO_3_ increasing from November 2021 to March 2022. Monitoring nitrate by colorimetry (Fig. 3A), the most obvious effect was the decrease in concentration from February and March 2022. The decrease in signal intensity and hence in concentration was caused by instable reagents (which are time- and temperature-dependent). During the whole investigation period, reagent solutions were prepared manually and provided freshly every 3 weeks or whenever analytically needed. A more frequent reagent replacement would be analytically beneficial but opposes the idea of running the instrument without manual interference. Dedicated to automated measurements, the device was usually unattended during monitoring, and manual work was reduced as much as possible. Manual quality controls for colorimetry were conducted every time new solutions were provided (cf. section “Materials and methods”) and compared to the results of online IC. Automated quality control routines and processes were not embedded by the manufacturer. Due to this reason, a comparison of colorimetric results from the end of February to the end of March was not possible. However, the reagent stability is a very important factor when it comes to the overall maintenance costs (cf. below). For the rest of the monitoring period from November 2021 to January 2022, analyses by colorimetry and optical sensor match well (± 0.25 mg∙L^−1^-NO_3_ in average, which means a relative 2.2% deviation). Moo et al. (2016) also employed optical fiber sensors for nitrate and nitrite in comparison to colorimetry. They found no interferences for nitrate as well as nitrite in lake, sewage, and leachate waters when measuring at the wavelengths 302 and 356 nm. Comparing the sensor and online-IC data, the average difference was 0.44 mg∙L^−1^-NO_3_ for the whole monitoring period (Fig. 3A) which is less than 5%. This level of difference can be considered tolerable for unattended online monitoring systems.

Nitrite concentrations over time at the river Rhine rarely reached 0.1 mg∙L^−1^-NO_2_ during the monitoring period from November 2021 to March 2022 (Fig. 3B). Total nitrate concentrations measured by online-IC differ about 12.5% and 16.3% from measured concentrations by sensor and colorimetry, respectively (i.e., absolute deviations of 1.2 and 1.6 mg∙L^−1^-NO_3_). These absolute and relative deviations per month are provided in SI 6. The drawback of high-frequency 24/7 analysis is the continuous injection of impurities into the systems, which can accumulate over time without regular and intensive cleaning procedures. Thus, when applied as online techniques and considering the 24/7 monitoring and automated quality control checks, a larger tolerance of up to 15% was applied for the online-IC compared to laboratory conditions where longer down times are acceptable (see section “Material and methods”). However, analysis by offline colorimetry using the same technique yielded identical results as by online-IC. Moreover, the analysis of spiked Rhine water showed a max. 15% deviation of sensor and IC results, which falls within the recovery tolerance range of quality control standards. Deviations between both techniques were on average 14%.

When comparing different automatic monitoring techniques, it is essential to consider not only technical aspects but also economic factors. This includes evaluating all costs such as initial costs and running costs, which include staff costs for maintenance and costs for consumables to ensure an uninterrupted 24/7 operation of the online monitoring systems. Last but not least, the provision of the specific infrastructure needs for each analytical technique has to be included in such calculations. Some factors during the comparison of the monitoring devices are difficult to convert into financial equivalents. These considerations include information gain from different analytical frequencies and the number of parameters analyzed by a single analytical system. In our comparison, the online-IC was clearly identified as the system with the highest initial costs (Table 2), estimated to be around 120,000 € (gross) along with the highest infrastructure demands due to its automated laboratory system nature. It needs dry and non-freezing conditions, no wind (e.g., also by air-condition), an ultrapure water generation system ideally directly connected to the IC, and for analysis filtered river water samples. However, these additional initial costs proved to be amortized over time due to its high level of automation, minimal maintenance time, analytical robustness, and analysis of a larger number of parameters (7 in this case), a temporal resolution of 30 min, and no chemical waste generation. These factors contribute to the long-term cost-effectiveness of the online-IC system. Another system used is the colorimetric device, initially significantly cheaper (Table 2) with an estimated initial cost of 25,000 € (gross) for a system capable of measuring nitrate and nitrite. However, the device incurred much higher costs for the consumable chemicals. Additionally, manual work for maintenance and operation was much higher with > 10 h/month. During the monitoring comparison, the sensor ex situ was the device with the least maintenance needs, followed by the sensor in situ with a higher need for manual maintenance like cleaning due to the biological activity in the natural system “river water” despite the sensors’ robustness. Additionally, infrastructure was needed for the sensor ex situ (a flow-through basin), while the sensor in situ just needed some infrastructure to be submerged into the river water. In terms of maintenance, the instruments can consequently be ordered in the following manner: sensor ex situ < sensor in situ < IC < colorimetry.

**Table 2:** Economic considerations in comparison of analytical online techniques

		IC	Colorimetry	Sensor
**Infrastructure**	Filtered river water	0.2 µm	< 1 µm	-
	Weather protection	Yes	Yes	-
	Ultrapure water	Yes	Yes	1×/year
	Number of anion analytes	7	2	1
	Amount of chemicals needed	11	8	-
	Chemical waste	-	yes	-
**Online feasibility**	Exchange of consumables	~ 1/month	1/month	-
	Handling time demand to generate consumables	1–2 h	~ 10 h	-
	Error proneness	Low	High	Low
	Error reports	Accurate	None	Accurate
	Min. analyses frequency	30 min	1 h	1 min
	Maintenance frequency	4–6 weeks	1×/month	2×/month
**Costs**	Approximate initial costs	120 k€	25 k€	12 k€
	Working time/month	⌀ 8 h	> 96 h	⌀ 30 min

However, it should be noted, once again, that the sensor covers, based on our results at the Rhine, only three parameters (nitrate and DOC_eq_ and TSS_eq_, which is used for correction of the other two analytes), whereas the online-IC covers 7 anions (F^−^, Cl^−^, Br^−^, SO_4_^2−^, PO_4_^3−^, NO_2_^−^, and NO_3_^2−^) and potentially could cover even more parameters like cations with the addition of a 2nd column (Wang et al., 2024). On the other hand, the colorimetric device covers a maximum of two parameters in a single device (in our case, nitrate and nitrite).

Summing up these considerations, all three devices reach comparable analytical results at different maintenance intensities and initial and running costs. On the one hand, the initial costs of the online–IC and the required infrastructure are high. On the other hand, using an online–IC as a single instrument, these costs enable a high degree of automation with minimal maintenance requirements, reduced staff costs, and more options on the analyte set. If no infrastructure is available onsite, the sensor is a good option with very low maintenance requirements for a limited set of analytes.

### Anion high-resolution time series

Using online-IC, the seven anions fluoride, chloride, bromide, nitrate, nitrite, phosphate, and sulfate were analyzed in the river Rhine at Koblenz for 2.5 years from December 2020 to May 2023. The monitoring is still ongoing and can be accessed by the link provided in materials and methods - time series. Additionally, the discharge was monitored by and received from the German Water and Shipping Administration. All data of this section are provided in the data repository (Appendix 3 and 4 zenodo.org/records/15736566.).

**Fig. 4:**
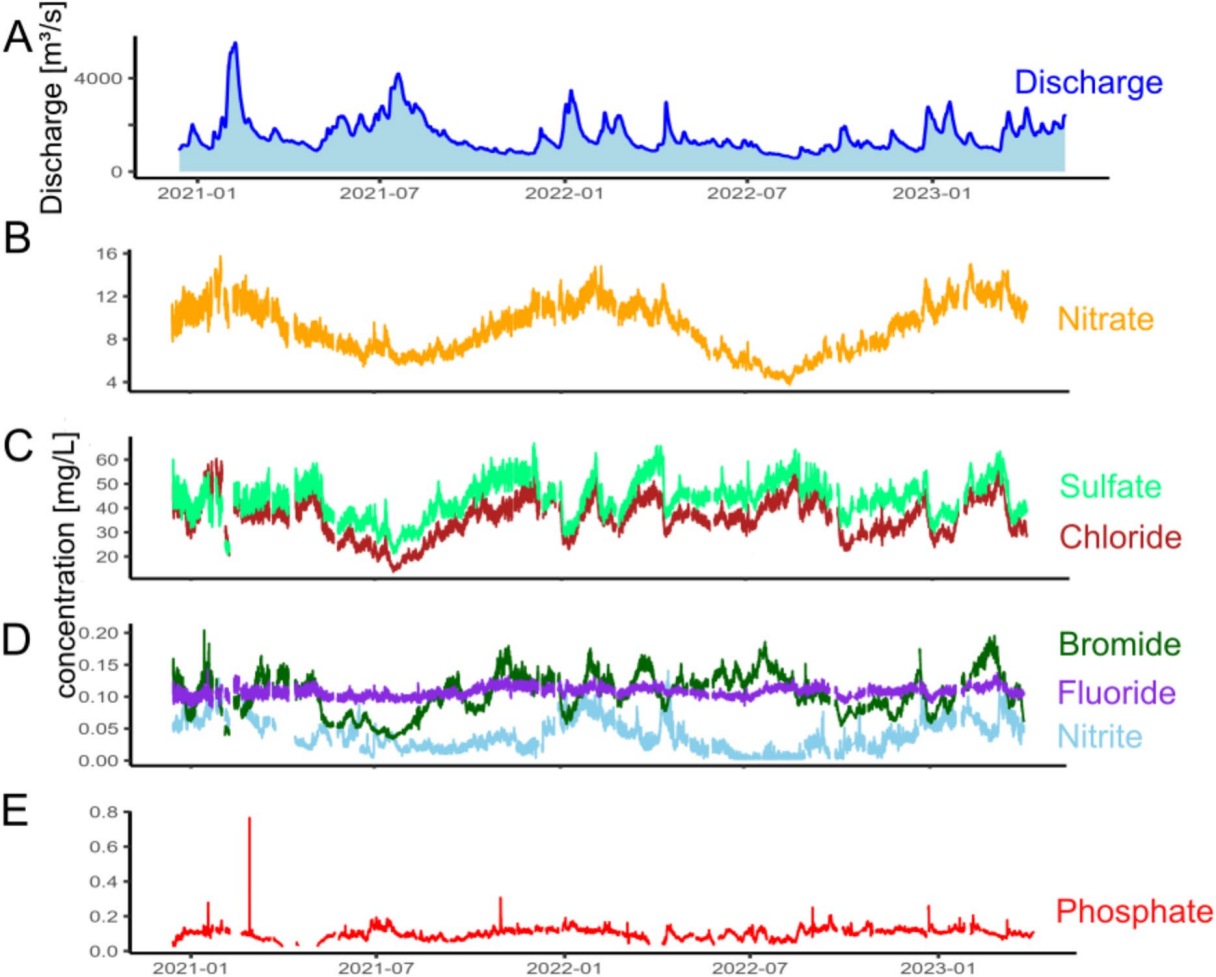
Time series of discharge and anion concentrations in mg·L^−1^ from December 2020 to March 2023 (mg∙L^−1^-NO_3_ for nitrate, mg∙L^−1^-PO_4_ for phosphate and mg∙L.^−1^-NO_2_ for nitrite)

In general, discharge was high during snowmelt every spring, and there was also a notable increase of discharge during the rainy summer of 2021, which resulted in flood events in various rivers in western Europe (Lehmkuhl et al., 2022). Periods with lower discharge occurred during autumn 2021 and especially summer to autumn 2022 (Fig. 4A). Continuous monitoring of anion concentrations was conducted, with the exception of some downtime periods related to pump operations (cf. Materials and methods—“Monitoring setup” section).

Time series of nitrate and nitrite generally exhibited a seasonal trend corresponding to the vegetation cycle. Nitrate concentrations were higher during winter, reaching levels of up to 14 mg∙L^−1^-NO_3_, while nitrite concentrations reached 0.14 mg∙L^−1^-NO_2_. In contrast, both nitrate and nitrite concentrations were lower during the summer, with nitrate concentrations averaging around 3.3 mg∙L^−1^-NO_3_ and nitrite concentrations at approximately 0.001 mg∙L^−1^-NO_2_ (Fig. 4B, D). This seasonal trend for nitrate concentrations aligns with long-term monitoring data collected from 219 different catchments across France from 11 to 2500 km^2^ with monitoring resolution of every 2 months (Minaudo et al., 2019). It also aligns with results reviewed from Burns et al. (2019), who monitor nitrate mainly by UV–Vis sensor in intervals < 1 h. The EU commission identifies fertilizers used in agriculture as the main source for nitrate in water, which enters rivers by runoff from agricultural fields (https://environment.ec.europa.eu/topics/water/nitrates_en).

At the river Rhine, sulfate concentration varies between 63 mg·L^−1^ during times of low discharge and 19 mg·L^−1^ in times of high discharge events, with a median concentration of 44 mg·L^−1^. A similar pattern is observed for chloride, with concentrations ranging between 55 and 12 mg·L^−1^ and a median of 33 mg·L^−1^. For bromide, the concentrations range from 0.19 to 0.03 mg·L^−1^ with a median concentration of 0.099 mg·L^−1^. These anion concentrations are strongly influenced by river discharge (Fig. 4C, D). An increase in river discharge leads to a decrease in concentration due to dilution. The negative correlation of discharge and chloride concentration was also shown at a southeastern New York rural stream for chloride by monthly sampling (Kelly et al., 2019), where the effect of road salting on the rivers was investigated. Additionally, the negative correlation was shown for unfiltered water samples from the river Rhine at Koblenz analyzed by ICP-MS (Belkouteb et al., n.d.) for the elements bromine and sulfur, which are predominantly present as bromide (Flury & Papritz, 1993; Gilfedder et al., 2011) and sulfate (Moore, 1991) in natural waters.

Fluoride concentration rarely experiences any changes over time and only slightly reacts to changing discharge (Fig. 4D; 0.09 ± 0.0065 mg·L^−1^). This can be attributed to its equilibrium with Ca and CaF_2_ in the river. CaF_2_ has a maximal solubility in water of 15 mg·L^−1^ and does rarely re-dissolve in water (Budavari, 1996).

The phosphate concentration shows a high short-term variability with an increase in river discharge. In these cases, the concentration increased by a factor of 7, with a median of 0.1 mg∙L^−1^-PO_4_ reaching maximum concentrations of > 0.7 mg∙L^−1^-PO_4_ (Fig. 4E). One explanation for this is the combined sewer overflow located about 500 m upstream of the monitoring station. During and after local rain events, discharges of rain-water combined with sewage into the river. With its proximity of only 500 m upstream of the monitoring station, the mixing of sewage with river water may not be completed when reaching the monitoring station.

**Fig. 5:**
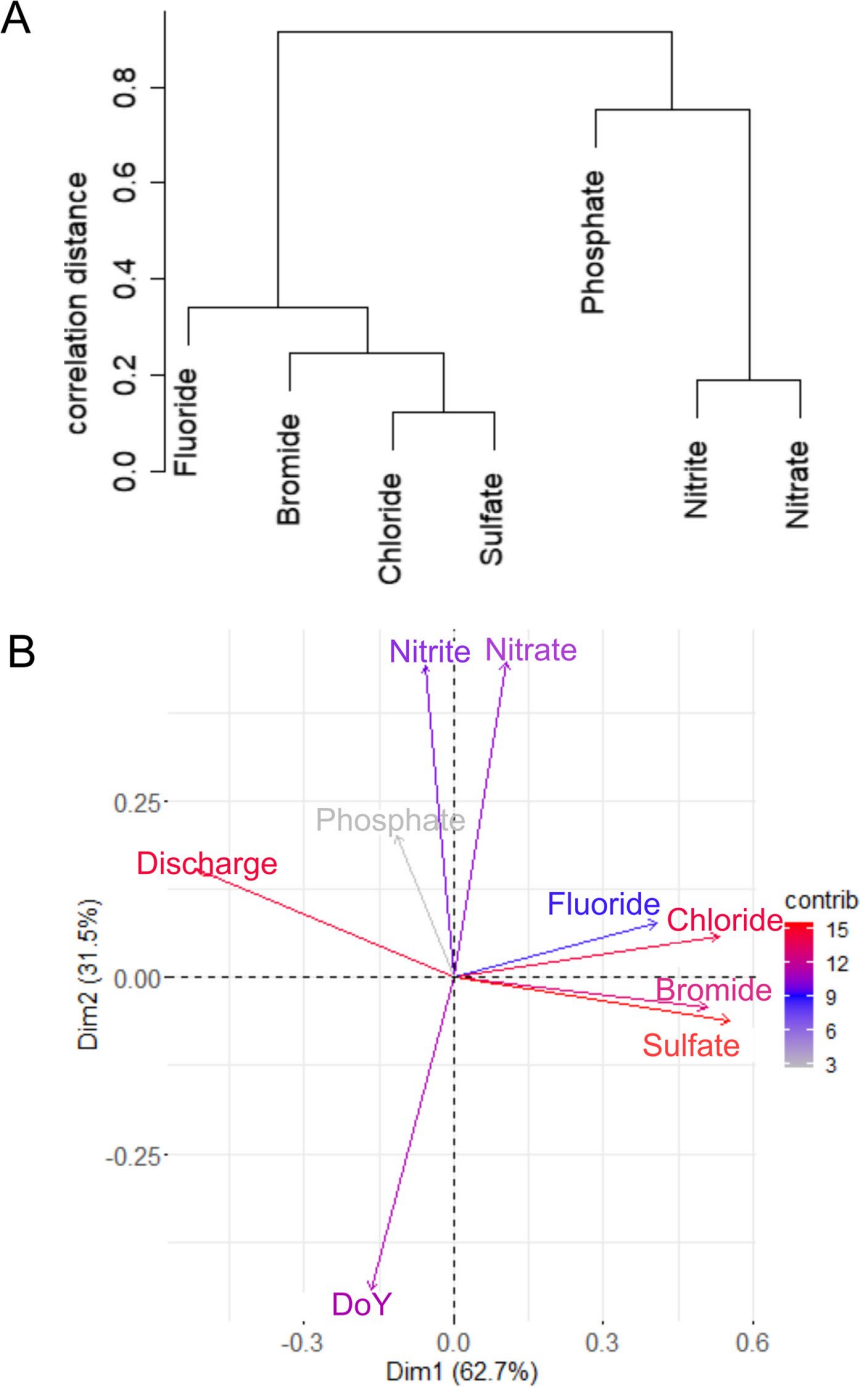
**A** Correlation dendrogram of analyzed anions for the whole monitoring period from December 2019 to March 2023 and **B** Principal Component Analysis (PCA) including additionally the two parameters discharge and “seasonality” (DoY)

Similarity of temporal element patterns becomes visible by clustering the elements using the average clustering method and a distance calculation by correlation. Three major groups are identified by the dendrogram: a group consisting of chloride and sulfate which is similar to bromide but has more distant similarities to fluoride, a second group containing the nutrients nitrate and nitrite, and a third group consisting of phosphate, which has distant similarities to the latter group but forms its own group (Fig. 5A). These groups support the similarities found examining time series data (Fig. 4) with chloride, sulfate, and bromide where a decrease in concentration is caused by an increasing discharge. For fluoride, this pattern is existent, but rarely visible at its low concentration range. With higher discharge, calcium concentration also decreases in the unfiltered water sample of the river Rhine (Belkouteb et al., Submitted), but as a major cation, it is still available in excess to fluoride and consequently still precipitates. The similarity for the group of nitrate and nitrite is the seasonal trend (Fig. 4). Phosphate forms a separate group by itself, which partly seems to be connected to the latter two anions, as it also is a nutrient that is applied in fertilizers and affected by biologic activity.

A principal component analysis shows again the similarity of element patterns (Fig. 5B). The contribution of the parameters becomes visible (by color), but also the interdependencies of the parameters (by direction). As an example, discharge and sulfate exhibit a negative correlation, as well as discharge and chloride or bromide (dilution of the present concentration). This correlation is weaker for fluoride, also due to its Ca-dominated behavior. There is no correlation for phosphate. Nitrate and nitrite are negatively correlated to the “day of the year” parameter (DoY, cf. Data evaluation–“Time series” section). With increasing DoY (from January to June), nitrate and nitrite decrease, and vice versa, reflecting the vegetation period with biologic activity in rivers and the agriculture period.

**Fig. 6:**
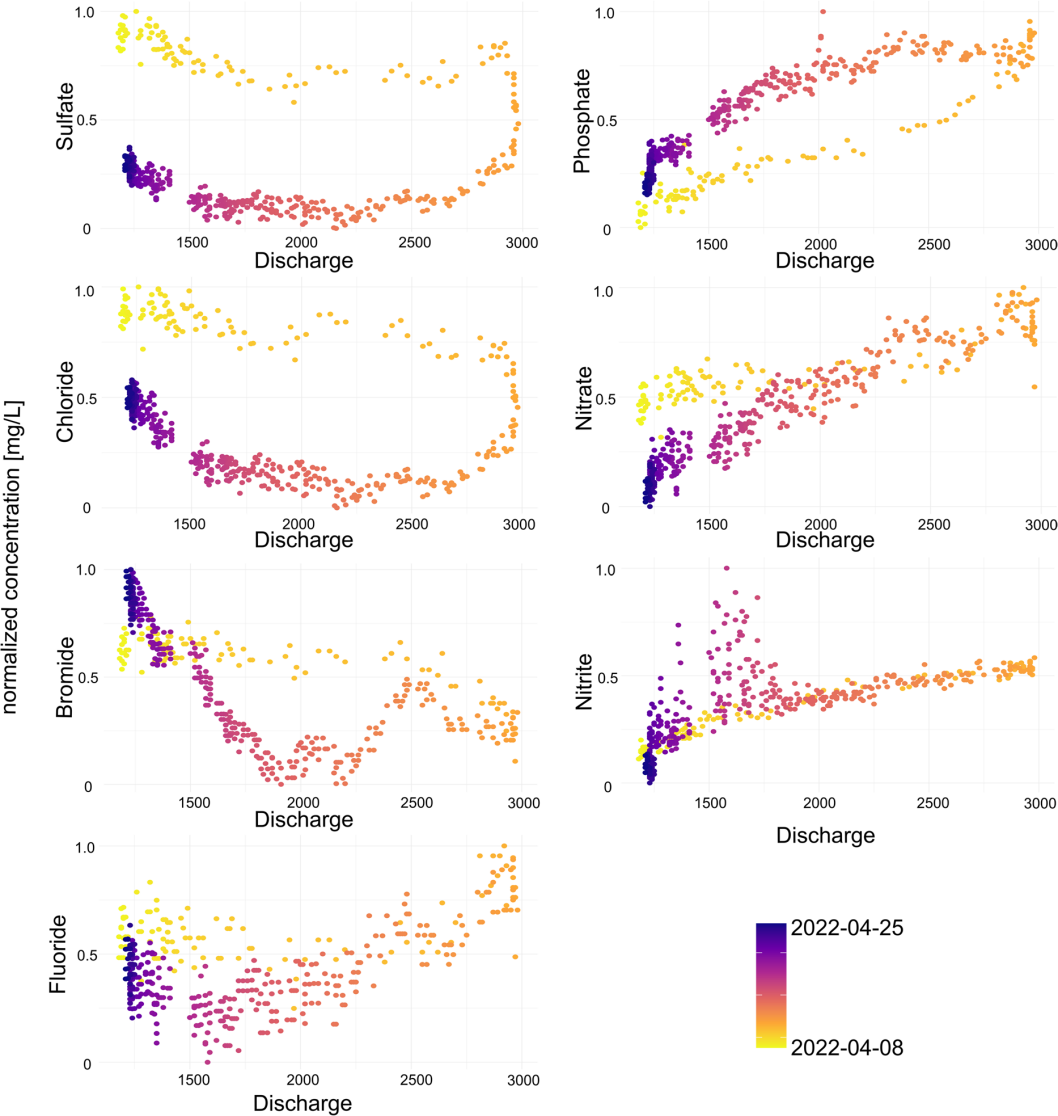
QC diagrams of all anions monitored for the peak during April 8 to 25, 2022, during a spring flood event. Colors indicate the date. Concentrations were normalized to the maximal concentration of each anion to ensure comparability of the analytes

During the monitoring period, there were several flood events. A closer look at the anion concentrations during these events reveals some insight on an anions’ origin and mobilization characteristics (e.g., Wang et al. (2024)). The different trends of the anion concentrations are visible during these flood events in the concentration – discharge plots (QC-plots, Fig. 6, Figs. SI 7, SI 8, SI 9), and as an example, the flood event from April 8 to 25, 2022, was chosen here. The same grouping of the anions is visible again. Sulfate, chloride, and bromide decrease in concentration with rising discharge and decrease to lower concentrations than before during discharge decrease. The fluoride concentration remains stable during the rising limb of the discharge and follows the same trend of the aforementioned group during decreasing discharge. In contrast, concentrations of phosphate, nitrate, and nitrite increase with increasing discharge. When discharge decreases, the nutrients phosphate, nitrate, and nitrite first increase in concentration before decreasing as well. This is congruent with the data above and also with findings by Wang et al. (2024), who monitored three different rivers in France by an online-IC for the selected anions chloride, sulfate, and nitrate. Bieroza and Heathwaite (2015) stated that substances with a QC-plot turning clockwise, such as sulfate, chloride, bromide, and fluoride in this study, have a hydrologic control which in our case would be the geogenic origin of these anions and a fast response to flood events (in this example by dilution in the river with surface runoff water). An anti-clockwise behavior, like observed for phosphate, nitrate, and nitrite at this flood event, marks the control by biogeochemical cycles in the catchment (in this example by additional analyte input via surface run-off and combined sewer overflow). This is another indication for grouping the anions analyzed in this study and is in line with the aforementioned results (Fig. 5).

Summing up, we evaluated the anion time series, performed PCA analysis, conducted clustering, and analyzed flood events. It can be concluded that on the one hand, sulfate, chloride, and bromide are affected by dilution during flood events and are mainly derived from geogenic sources, surface runoff, and treated waste water. On the other hand, nitrate and nitrite first increase in concentration before being diluted, indicating their origin from agriculture and waste water overflows. A comparable pattern is also visible for phosphate, which in this study is a special case due to the presence of a combined sewer outfall 500 m upstream of the monitoring station delivering a clear indication of the impact of untreated waste water. Additionally, the nutrients experience an underlying seasonal dynamic. Fluoride, in equilibrium with CaF_2_ in the river, exhibits a limitation in concentration but still follows the trend of a dilution-affected anion during flood events.

Data of the online–IC, which are verified and corrected according to Materials and methods—“Implementation of the online–IC” section, are provided in the data repository (Appendix 3 zenodo.org/records/15736566.). Additionally, data of the discharge for the same period are provided for Koblenz in the data repository (Appendix 4 zenodo.org/records/15736566.).

### Impact of time resolution on peak identification and load calculations

**Fig. 7:**
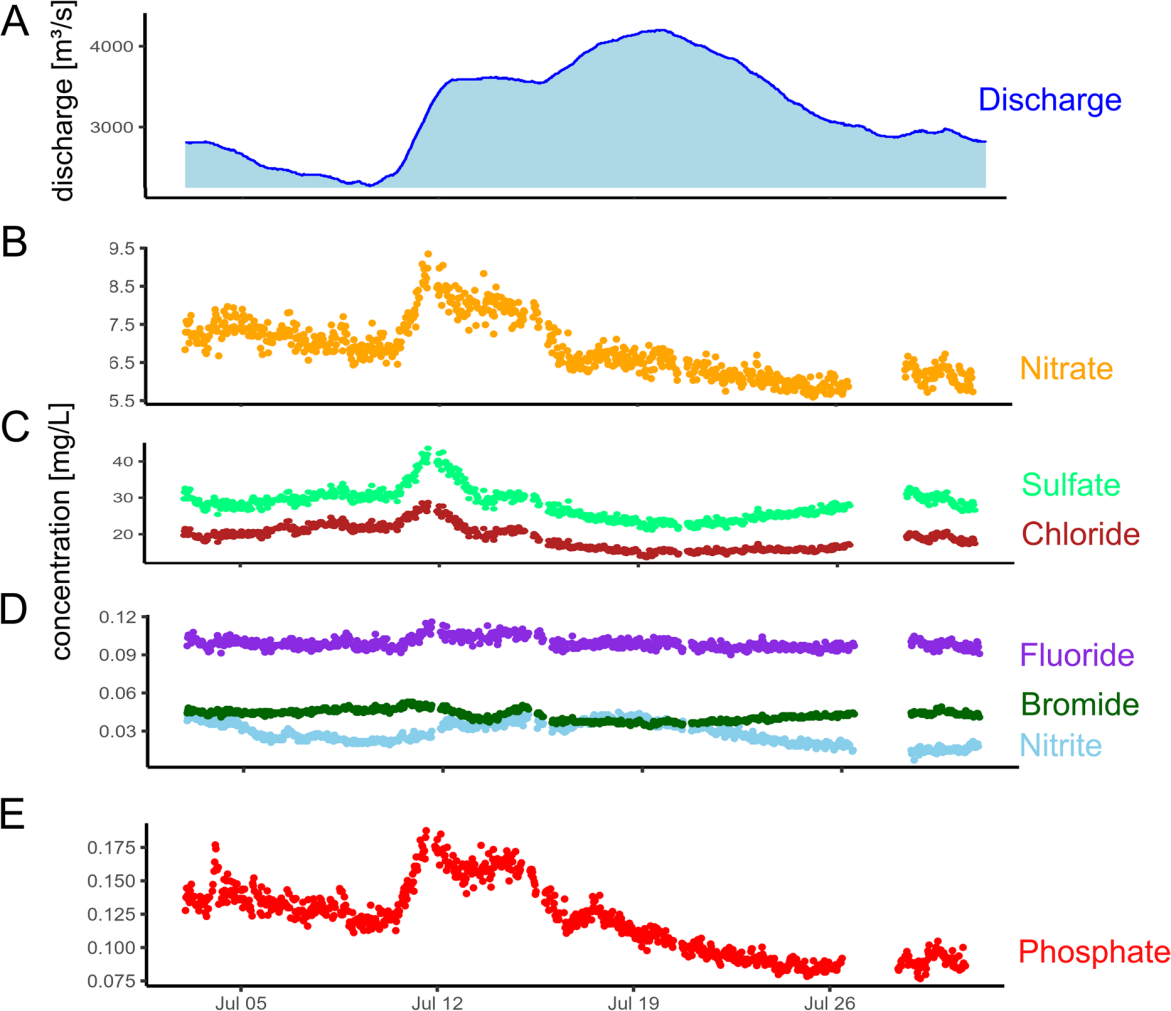
Continuous monitoring of anions and discharge during the flood event and following time of higher discharge in July 2021

Analyzing 40 samples per day, short-term changes become detectable by online–IC. Routine monitoring often involves manual grab or mixed sampling in a larger time interval, e.g., a grab sample every 14 days, as it is partly done at the river Rhine in Koblenz (ICPR, 2020). By their nature, larger time intervals fail to address potentially short-term peak concentrations and all short-term changes, as in July 2021 during a period of heavy rainfalls in western parts of Germany (Fig. 7). During the initial discharge increase (July 11th 2021), most of the anion concentrations (nitrate, sulfate, chloride, and nitrite) co-increase. However, during the further discharge increase, anion concentrations decrease again and are even lower than before the discharge event (dilution effect). Corresponding to Results and discussion—“Anion high-resolution time series” section, the reasons can be attributed to the initial washout of the anions from soils and urban surfaces (if separated sewers are in place) during the first flush event, while dilution takes place during the subsequent extended high discharge period. Similar trends with nitrate washouts during the first flush event have been seen for nitrate at Neuse River, NC, USA (Baker & Showers, 2019) and for monitoring of internal water storage (Donaghue et al., 2023), also in different climatic settings (Mastouri et al., 2023).

For the calculation of loads per month or year, the monitoring frequency is crucial. As an example, calculating loads using a 14-day grab sample monitoring interval in this case is based on sampling on 05.07.2021 and 19.07.2021 (ICPR, 2020), which does not cover the first concentration peak with moderately higher discharge on July 12th (Fig. 7). This results in an estimation of the total loads for the month around 100% ± 5% compared to the actual total loads calculated from the 30-min analyses by online-IC (Table SI 3). Surprisingly, the 14-day sampling interval also meets the average loads (93–100%; Table SI 4), which is in sharp contrast to the underestimation of the maximal concentrations for chloride (70%), nitrite (50%), nitrate (77%), phosphate (74%), and sulfate (67%; Table SI 5). The estimation and calculation of total and average loads of the rivers is important and frequently done based on low-frequency data, neglecting that extreme concentrations are most crucial for the river ecosystem and often extreme events are dominating the annual load budgets of rivers (Yevenes & Mannaerts, 2011). A low-frequency monitoring is important to capture long-term changes and is economically feasible at several locations. This was also found by Bieroza et al. (2014), who analyzed the nutrients nitrate, total reactive phosphorus, and total phosphorus in an agricultural catchment at two rivers in northern Great Britain. For high-frequency monitoring, however, short-term concentration peaks can set the lower limit of a reasonable monitoring interval.

**Fig. 8:**
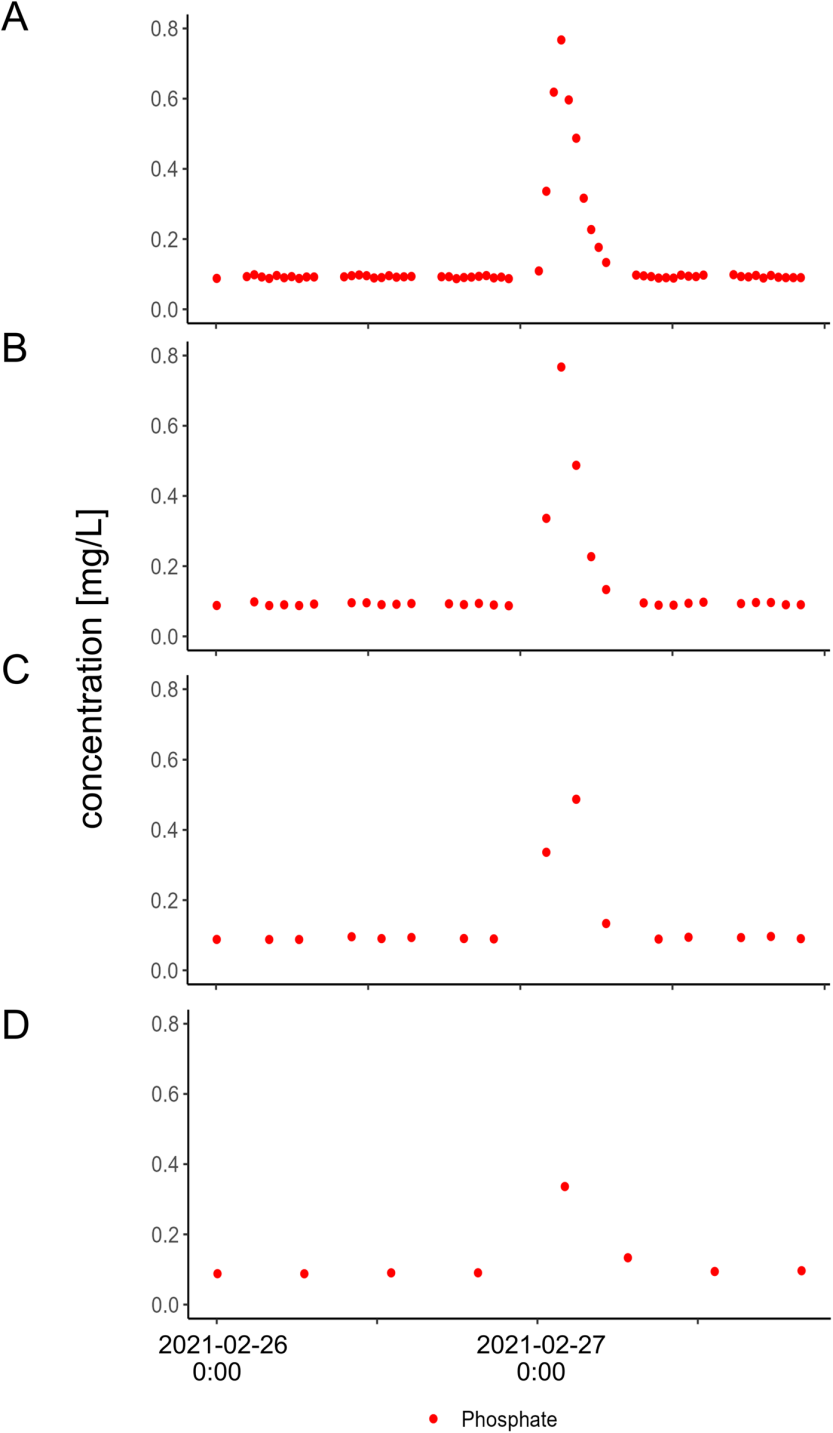
Different time resolution for the shortest peak which occurred in February 2021 for phosphate. Time intervals shown are **A** 30 min, **B** 1 h, **C** 2 h, **D** 4 h

During the entire monitored period, most anion peaks were several days long (cf. Fig. 4). Phosphate was the only anion that exhibited short steep peaks down to total durations of 5 h. Presumably, these short peaks with durations as short as hours are caused by a mixed water overflow basin inflow approximately 500 m before the monitoring station (cf. Results and discussion–“Anion high-resolution time series” section). We coarsened our sampling interval for our shortest phosphate peak we monitored to show how easy it can be misinterpreted as an outlier when sampling intervals are extended (Fig. 8).

The Rhine is a less likely fluctuating system compared to a stream with a lower discharge and smaller catchment. An even smaller time interval might be needed for more dynamic (smaller) streams. Smaller streams often show a larger temporal dynamic in most parameters, starting from discharge leading to turbidity and transport of substances and elements. To capture a 5-h peak, time intervals of at least every 2 h are suggested (Fig. 8). For peaks with a longer duration, larger time intervals might be sufficient. Similar results have been found by Neal et al. (2012), who monitored the river Severn (UK) and found that a frequency of 7 h is not sufficient to reveal chemical dynamics like diurnal concentration changes for nitrate during low-flow periods and to capture the entire rising limb of a hydrograph in the case of a flood event. However, the Severn, with its mean discharge of 107 m^3^·s^−1^, cannot fully be compared to the Rhine, which has a mean discharge of 2000 m^3^·s^−1^. The Rhine is a less likely fluctuating system compared to a stream with a lower discharge and smaller catchment. An even smaller time interval might be needed for more dynamic (smaller) streams. Wang et al. (2024) monitored three rivers in France every 7 h for anions and cations and found solutes like chloride and sulfate, which were diluted by storm events, were overestimated, while nutrients like nitrate, which are mobilized by the storm, were underestimated. These findings are congruent with our results. Wang et al. (2024) found this effect to be most obvious in large rivers and to be most likely if “storm events” are missed during the set sampling interval.

Summing up, a correct estimation of mean, total, and maximum anion loads is important and depends on the stability of the system and the monitoring interval. In our example, total and mean anion loads were calculated close to correct during the discharge event although monitoring was done every 2 weeks and did not capture the peak discharge and peak concentrations. However, maximum concentrations were by far underestimated. Shortest concentration peaks due to close-by point sources need to be considered during the selection of the monitoring location and intervals. In our case, phosphate is now used to monitor the potential impact of the combined sewer overflow close by and was initially used for the identification of the required time resolution of all other at- and online analyses.

## Conclusions

Real-time high-frequency monitoring gains increasing attention in environmental monitoring due to several pressures (Arndt et al., 2022). We employed and evaluated three real-time monitoring techniques at the river Rhine in Koblenz and additionally analyzed anion time series for 2.5 years. The evaluated techniques were (1) a sensor for nitrate in situ and ex situ, (2) a colorimetric device for nitrate and nitrite, and (3) an online ion chromatography (IC) for the seven anions fluoride, bromide, chloride, nitrate, nitrite, phosphate, and sulfate. Evaluation of the monitoring techniques resulted in no difference for the chosen monitoring location for nitrate in situ (in the river) and ex situ (in a bypass to a monitoring station) in contrast to DOC_eq_, which is more sensitive to turbidity and fouling and needs significantly more frequent maintenance effort (i.e., regularly manual cleaning). Comparing the online-IC, the sensor, and the colorimetric device, the techniques need more maintenance in ascending order. The analytical results are in the same order of magnitude, as long as the specific amount of maintenance for each instrument is provided, which in some cases contradicts the automatization aim. The initial costs, however, decrease in this order. The degree of automation is very high for the online-IC, lower for the sensor, and even lower for the colorimetric device. The number of analyzed elements is highest for the online-IC. Thus, when online anion monitoring is planned, we recommend implementing an online-IC if the respective infrastructure is in place. From the authors’ experience, the initial costs of the IC are amortized more quickly with lower maintenance and fewer chemicals. The list of analytes can easily be extended to cations.

Analyzing the high-frequency anion time series, it was possible to improve our understanding of the anion dynamics and to identify different underlying processes. The major groups of anions clustered by dendrogram, principal component analysis, and the plotting of QC diagrams were (1) the discharge-controlled anions sulfate, chloride, bromide, and fluoride, with the predominant sources of surface runoff and treated waste water, whereas fluoride is also controlled by equilibrium precipitation, and (2) the anions nitrate, nitrite, and phosphate driven by seasonal dynamics as well as agriculture run-off, and untreated waste water from a combined sewer overflow, with the last aspect being most visible for phosphate. Our findings provide actionable insights for river basin managers to design early-warning systems, to safeguard drinking water intakes, to refine load calculations. The recommended high-frequency monitoring by online IC can, for instance, help to define the minimum monitoring frequency needed by identifying the shortest occurring peaks in the monitoring location. We defined the minimum monitoring frequency to be max. 2 h at the monitoring station Koblenz for the river Rhine. This allows us to capture short-term changes and to identify the impact from closed-by sources like combined sewer overflows and to improve annual budgets.

## Supplementary Information

Below is the link to the electronic supplementary material.

ESM 1 (DOCX 871 KB)

## Data Availability

Next to the supporting information provided with this manuscript, the full data set is provided via the repository zenodo.org/records/15736566 with the appendices 1–4.
