# Supplementary material for: Online high-resolution real-time monitoring techniques for anions in river water
Source: Environ Monit Assess. 2026 Jan 13;198(2):121. doi: 10.1007/s10661-025-14954-y (PMC12795966; doi:10.1007/s10661-025-14954-y)

**Online high-resolution real-time monitoring techniques for anions in river water**

Julia Arndt1,2, Anna-Lena Gerloff1, Alex Zavarsky1*, Michael P. Schlüsener1, Arne Wick1, Lars Duester1

1: Federal Institute of Hydrology, Am Mainzer Tor 1, 56068 Koblenz, Germany

2: North Rhine Westphalia Office for Nature, Environment and Climate, Germany

*zavarsky@bafg.de

**Supporting Information**

| SI 1 | Sequence of the ion chromatography in online analysis mode. |
| --- | --- |
| SI 2 | Calibration standards of online-IC in mg·L^‑ 1^_._ |
| SI 3 | Comparison of monthly total anion loads for 14-day analysis and continuous analysis (online-IC) in percent. |
| SI 4 | Comparison of monthly mean anion loads for 14-day analysis and continuous analysis (online-IC) in percent. |
| SI 5 | Comparison of monthly maximum anion loads for 14-day analysis and continuous analysis (online-IC) in percent. |
| SI 6 | Differences between analytical techniques per month. |
| SI 7 | Discharge and concentration during the flood event from 20^th^ December 2022 to 9^th^ January 2023. |
| SI 8 | Discharge and concentration during the flood event from 28th September 2022 to 14^th^ October 2022. |
| SI 9 | Discharge and concentration during the flood event from 9^th^ January 2023 to 31^st^ January 202 |
| SI10 | A normalized version of Figure 2 |

SI 1: Sequence of the ion chromatography in online analysis mode. 10 river water samples were analyzed. After 10 samples, a blank sample (ultrapure water) was analyzed followed by two calibration check standards. If these met the recoveries of 85-115% for the anions fluoride, bromide, chloride, nitrate, phosphate, and sulfate, the next ten samples were analyzed. If recoveries were not met for one of the anions, a re-calibration was done with two subsequent calibration check standards. If these two met recoveries, the next ten samples were analyzed. If recovery was not met after re-calibration, 100 river samples were analyzed (until manual intervention). Phosphate was the only anion which did not induce a re-calibration when recoveries were not met and was monitored only.


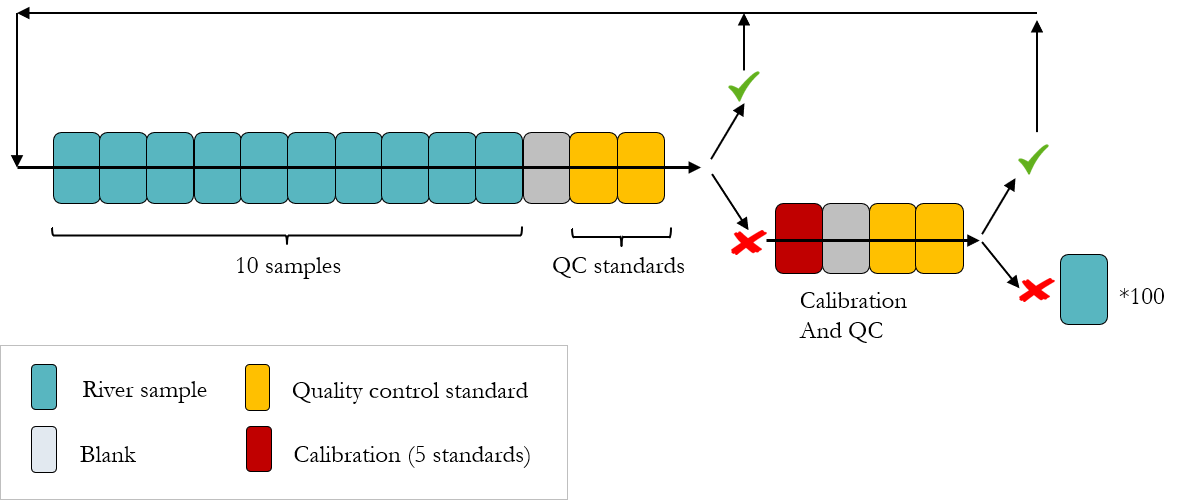


SI 2: Calibration standards of online-IC in mg·L^‑ 1^. Calibration was adjusted to these ranges based on the monthly samples analyzed during the IKSR analysis program from previous years.

| Parameter | Cal 1 | Cal 2 | Cal 3 | Cal 4 | Cal 5 |
| --- | --- | --- | --- | --- | --- |
| Fluoride | 0.05 | 0.1 | 0.25 | 0.4 | 0.5 |
| Chloride | 10 | 25 | 50 | 75 | 100 |
| Nitrite | 0.01 | 0.025 | 0.05 | 0.75 | 0.1 |
| Bromide | 0.05 | 0.1 | 0.25 | 0.4 | 0.5 |
| Nitrate | 2 | 5 | 10 | 15 | 20 |
| Phosphate | 0.05 | 0.1 | 0.25 | 0.4 | 0.5 |
| Sulfate | 10 | 25 | 50 | 75 | 100 |

SI 3: Comparison of monthly total anion loads for 14-day analysis and continuous analysis (online-IC) in percent. Values >100% (marked in blue) indicate an overestimation of loads for a 14-day analysis while values <100% indicate an underestimation (marked in red).

| Date | Fluoride | Chloride | Nitrite | Bromide | Nitrate | Phosphate | Sulfate |
| --- | --- | --- | --- | --- | --- | --- | --- |
| 2020-12 | 72.4 | 75.6 | NA | 79.8 | 66.7 | 59.7 | 69.4 |
| 2021-01 | 110.6 | 104.8 | 93.5 | 114.1 | 102.2 | 101.4 | 113.3 |
| 2021-02 | 161.8 | 222.7 | NA | 178.9 | 177.7 | NA | 178.1 |
| 2021-03 | 106.4 | 112.2 | 160.9 | 98 | 113.4 | 133.5 | 108.5 |
| 2021-04 | 121.5 | 124.8 | 340 | 124.9 | 136 |  | 130.9 |
| 2021-05 | 118 | 125.7 | 127.6 | 122.6 | 116.9 | 86.4 | 124 |
| 2021-06 | 98.9 | 103.9 | 111.1 | 108 | 97 | 78.5 | 98 |
| 2021-07 | 97.5 | 98.9 | 96.3 | 103.7 | 100.4 | 97.2 | 96.1 |
| 2021-08 | 94.6 | 90.1 | 69.6 | 82.9 | 93.2 | 96.4 | 93.3 |
| 2021-09 | 95.9 | 97.5 | 103.6 | 100 | 96.3 | 102.9 | 96.4 |
| 2021-10 | 94.4 | 88.8 | 57 | 95.6 | 90.9 | 84.6 | 90.4 |
| 2021-11 | 102.4 | 95.9 | 112.3 | 101.8 | 101.4 | 105.3 | 102 |
| 2021-12 | 120.2 | 127.7 | 105.3 | 119 | 117.4 | 125.1 | 135.7 |
| 2022-01 | 99.5 | 101.1 | 85.2 | 99.5 | 95.4 | 94.4 | 96.5 |
| 2022-02 | 116.3 | 123.7 | 105.8 | 127.4 | 117.7 | 112.3 | 120.1 |
| 2022-03 | 98.6 | 93.8 | 113.2 | 90.5 | 101 | 138.2 | 92.4 |
| 2022-04 | 103.6 | 104.1 | 107.8 | 107.9 | 105.1 | 130.6 | 106 |
| 2022-05 | 127 | 123.4 | 138.4 | 123 | 127.4 | 116.5 | 123.9 |
| 2022-06 | 99.3 | 98.6 | 69 | 96.7 | 97.5 | 84.5 | 97.5 |
| 2022-07 | 101.1 | 97.1 | 69.1 | 103.6 | 101.3 | 86.2 | 102.9 |
| 2022-08 | 101.3 | 104.1 | 60.7 | 112.9 | 93.9 | 72 | 104.1 |
| 2022-09 | 117.1 | 118.1 | 87.9 | 123.2 | 117.3 | 108.8 | 119.6 |
| 2022-10 | 127.3 | 141.1 | 96.7 | 145.9 | 121.5 | 123.7 | 134.1 |
| 2022-11 | 100 | 100.7 | 145.9 | 98 | 98.7 | 108.5 | 103.9 |
| 2022-12 | 99.9 | 99.4 | 80.2 | 109 | 87.9 | 89.7 | 108.6 |
| 2023-01 | 110.1 | 109.8 | 87.7 | 110.4 | 112.6 | 115.5 | 108.7 |
| 2023-02 | 107.2 | 106.2 | 106.6 | 111.9 | 106.8 | 105.2 | 102.3 |
| 2023-03 | 110.1 | 116.5 | 101.4 | 122.8 | 115.9 | 102.9 | 124 |

SI 4: Comparison of monthly mean anion loads for 14-day analysis and continuous analysis (online-IC) in percent. Values >100% (marked in blue) indicate an overestimation of loads for a 14-day analysis while values <100% (marked in red) indicate an underestimation.

| Date | Fluoride | Chloride | Nitrite | Bromide | Nitrate | Phosphate | Sulfate |
| --- | --- | --- | --- | --- | --- | --- | --- |
| 2020-12 | 130.4 | 136.2 | NA | 143.1 | 120 | 107.4 | 124.9 |
| 2021-01 | 103.7 | 98 | 96.8 | 106.6 | 95.7 | 95.2 | 106 |
| 2021-02 | NA | 167 | NA | 134 | 133.3 | NA | 133.6 |
| 2021-03 | 99.4 | 104.9 | 119.5 | 91.7 | 106.1 | 124.7 | 101.5 |
| 2021-04 | 100.8 | 99.8 | 206.9 | 103.6 | 113.4 | 100 | 104.7 |
| 2021-05 | 106.1 | 113.6 | 106.7 | 110.7 | 105.6 | 72.6 | 112 |
| 2021-06 | 99 | 103.9 | 111.1 | 108.1 | 97 | 78.9 | 98 |
| 2021-07 | 94.4 | 95.7 | 92.9 | 100.7 | 97.1 | 94 | 93 |
| 2021-08 | 94.4 | 90.1 | 68.1 | 82.9 | 93.2 | 93.5 | 90.3 |
| 2021-09 | 95.6 | 97.5 | 104 | 100 | 96.3 | 100 | 96.4 |
| 2021-10 | 94.3 | 88.8 | 57.1 | 95.5 | 90.9 | 84.4 | 90.4 |
| 2021-11 | 100 | 92.7 | 108 | 98.3 | 98 | 102.1 | 98.6 |
| 2021-12 | 100.7 | 107.1 | 88.3 | 100 | 98.5 | 97.2 | 105 |
| 2022-01 | 99.6 | 101.1 | 84.9 | 99.5 | 95.4 | 94.6 | 96.5 |
| 2022-02 | 103.7 | 110.5 | 94 | 113.9 | 105.1 | 100 | 107.2 |
| 2022-03 | 98.4 | 93.8 | 111.1 | 90 | 101 | 107.4 | 92.4 |
| 2022-04 | 103.6 | 104.1 | 107.3 | 108.1 | 105.1 | 113.3 | 106 |
| 2022-05 | 102.3 | 99.5 | 114.3 | 98.7 | 102.8 | 93.7 | 99.9 |
| 2022-06 | 99.1 | 98.5 | 70.6 | 96.8 | 97.5 | 83.8 | 97.5 |
| 2022-07 | 97.9 | 94 | 100 | 100 | 98.1 | 82.7 | 99.6 |
| 2022-08 | 101.2 | 104.1 | 57.1 | 112.9 | 93.9 | 71.8 | 104.1 |
| 2022-09 | 97.1 | 98.4 | 73.9 | 103.2 | 97.7 | 90.6 | 99.7 |
| 2022-10 | 98.6 | 109.2 | 75.9 | 112.9 | 94.1 | 95.7 | 103.8 |
| 2022-11 | 100 | 100.7 | 145.5 | 99 | 98.7 | 108.3 | 103.9 |
| 2022-12 | 100 | 99.4 | 80.3 | 109.4 | 87.9 | 86.8 | 101.6 |
| 2023-01 | 92.4 | 92.1 | 73.5 | 92.7 | 94.4 | 96.8 | 91.1 |
| 2023-02 | 95.7 | 94.9 | 96 | 100 | 95.4 | 93.5 | 91.3 |
| 2023-03 | 106.8 | 112.7 | 98.1 | 118.8 | 112.1 | 100 | 120 |

SI 5: Comparison of monthly maximum anion loads for 14-day analysis and continuous analysis (online-IC) in percent. Values >100% (marked in blue) indicate an overestimation of loads for a 14-day analysis while values <100% (marked in red) indicate an underestimation.

| Date | Fluoride | Chloride | Nitrite | Bromide | Nitrate | Phosphate | Sulfate |
| --- | --- | --- | --- | --- | --- | --- | --- |
| 2020-12 | 91.7 | 87.7 | NA | 75.0 | 81.3 | 57.1 | 76.0 |
| 2021-01 | 85.7 | 91.9 | 76.9 | 70.0 | 92.5 | 46.4 | 89.2 |
| 2021-02 | 84.6 | 84.5 | NA | 90.0 | 79.3 | NA | 84.5 |
| 2021-03 | 91.7 | 91.3 | 87.5 | 81.3 | 93.7 | 83.3 | 80.0 |
| 2021-04 | 91.7 | 78.1 | 100 | 80.0 | 93.4 | 80.0 | 92.8 |
| 2021-05 | 91.7 | 63.4 | 83.3 | 50.0 | 87.3 | 46.7 | 76.8 |
| 2021-06 | 83.3 | 83.1 | 44.4 | 87.5 | 75.0 | 52.6 | 88.0 |
| 2021-07 | 83.3 | 70.6 | 50.0 | 100.0 | 77.0 | 73.7 | 66.9 |
| 2021-08 | 83.3 | 86.2 | 50.0 | 90.9 | 86.0 | 66.7 | 84.0 |
| 2021-09 | 83.3 | 84.8 | 66.7 | 85.7 | 84.7 | 63.6 | 79.0 |
| 2021-10 | 92.3 | 76.3 | 60 | 75.0 | 87.4 | 38.7 | 87.0 |
| 2021-11 | 92.3 | 81.6 | 80.0 | 88.9 | 89.2 | 75.0 | 89.3 |
| 2021-12 | 92.3 | 88.2 | 66.7 | 93.3 | 85.5 | 76.5 | 83.7 |
| 2022-01 | 92.3 | 91.3 | 66.7 | 83.3 | 90.2 | 68.4 | 91.3 |
| 2022-02 | 84.6 | 68.6 | 60.0 | 75.0 | 79.3 | 64.7 | 61.3 |
| 2022-03 | 91.7 | 85.8 | 55.6 | 88.2 | 89.1 | 62.5 | 90.3 |
| 2022-04 | 92.3 | 70.9 | 64.3 | 73.3 | 98.1 | 93.3 | 76.2 |
| 2022-05 | 100.0 | 89.4 | 66.7 | 85.7 | 90.2 | 66.7 | 91.4 |
| 2022-06 | 90.9 | 82.6 | 25.0 | 81.3 | 78.5 | 69.2 | 79.6 |
| 2022-07 | 91.7 | 79.9 | 0 | 84.2 | 73.6 | 53.3 | 79.8 |
| 2022-08 | 92.3 | 82.2 | 33.3 | 93.8 | 95.0 | 52.0 | 88.0 |
| 2022-09 | 84.6 | 78.6 | 37.5 | 84.6 | 82.3 | 57.1 | 80.3 |
| 2022-10 | 100.0 | 88.8 | 57.1 | 88.9 | 95.1 | 88.2 | 92.2 |
| 2022-11 | 91.7 | 71.9 | 83.3 | 90.0 | 88.1 | 86.7 | 90.7 |
| 2022-12 | 84.6 | 79.9 | 33.3 | 72.2 | 72.1 | 46.2 | 84.2 |
| 2023-01 | 91.7 | 74.0 | 50.0 | 83.3 | 94.0 | 73.7 | 87.6 |
| 2023-02 | 92.3 | 89.3 | 60.0 | 94.7 | 85.9 | 57.1 | 97.2 |
| 2023-03 | 100.0 | 81.1 | 70.0 | 81.3 | 108.0 | 71.4 | 90.4 |

SI 6: Differences between analytical techniques per month

|  | Month | Nitrate | | | Nitrite |
| --- | --- | --- | --- | --- | --- |
|  |  | IC vs. sensor | IC vs. colorimetry | Sensor vs. colorimetry | IC vs. colorimetry |
| Absolute difference  [mg·L^‑ 1^] | 2021-11 | 0.43 | 0.55 | 0.32 | 0.0095 |
|  | 2021-12 | 0.47 | 0.51 | 0.16 | 0.0033 |
|  | 2022-01 | 0.42 | 0.54 | 0.26 | 0.0035 |
|  | 2022-02 | 0.47 | 2.74 | 2.90 | 0.0082 |
|  | 2022-03 | 0.40 | 9.55 | 10.00 | 0.0071 |
|  | entire time | 0.44 | 1.14 | 0.99 | 0.0061 |
| Relative difference  [%] | 2021-11 | 4.4 | 5.7 | 3.1 | 29.2 |
|  | 2021-12 | 4.4 | 4.6 | 1.4 | 8.7 |
|  | 2022-01 | 3.8 | 4.8 | 2.2 | 4.1 |
|  | 2022-02 | 4.1 | 24.7 | 25.4 | 11.9 |
|  | 2022-03 | 3.8 | 89.9 | 90.4 | 14.3 |
|  | entire time | 4.1 | 10.4 | 8.7 | 13.8 |

SI 7: Discharge and concentration during the flood event from 20^th^ December 2022 to 9^th^ January 2023.


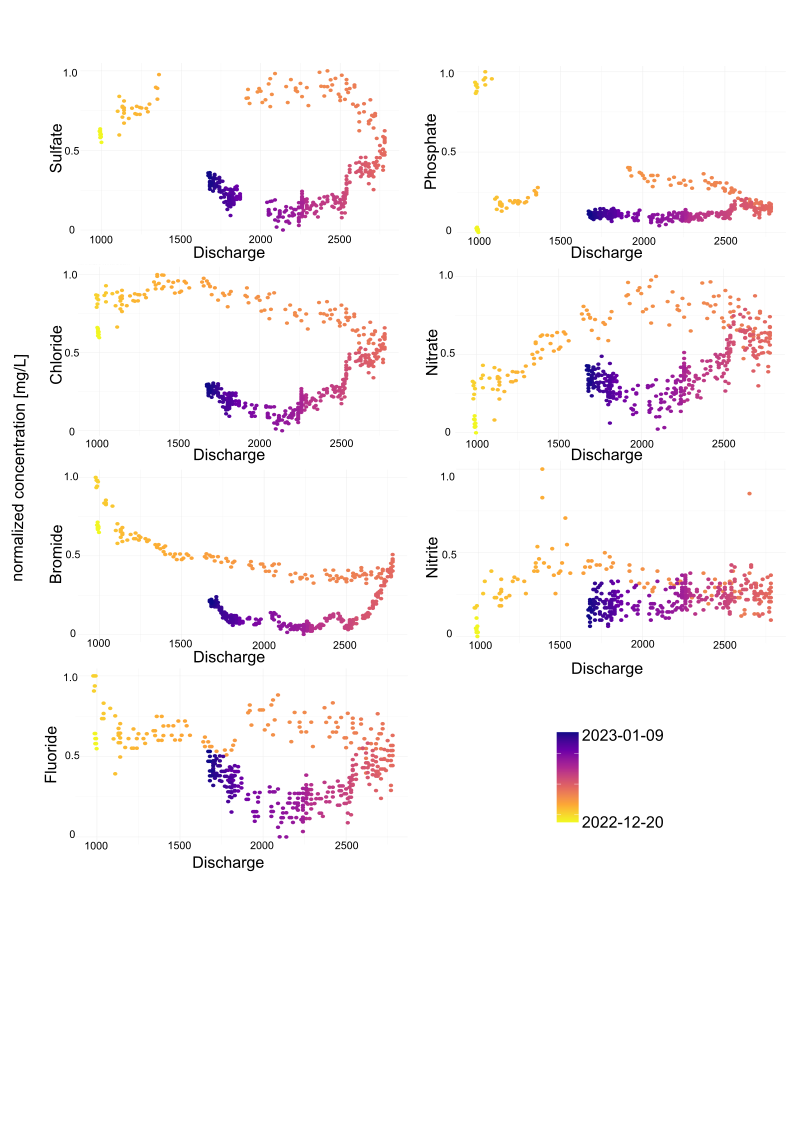


SI 8: Discharge and concentration during the flood event from 29th September 2022 to 14^th^ October 2022.


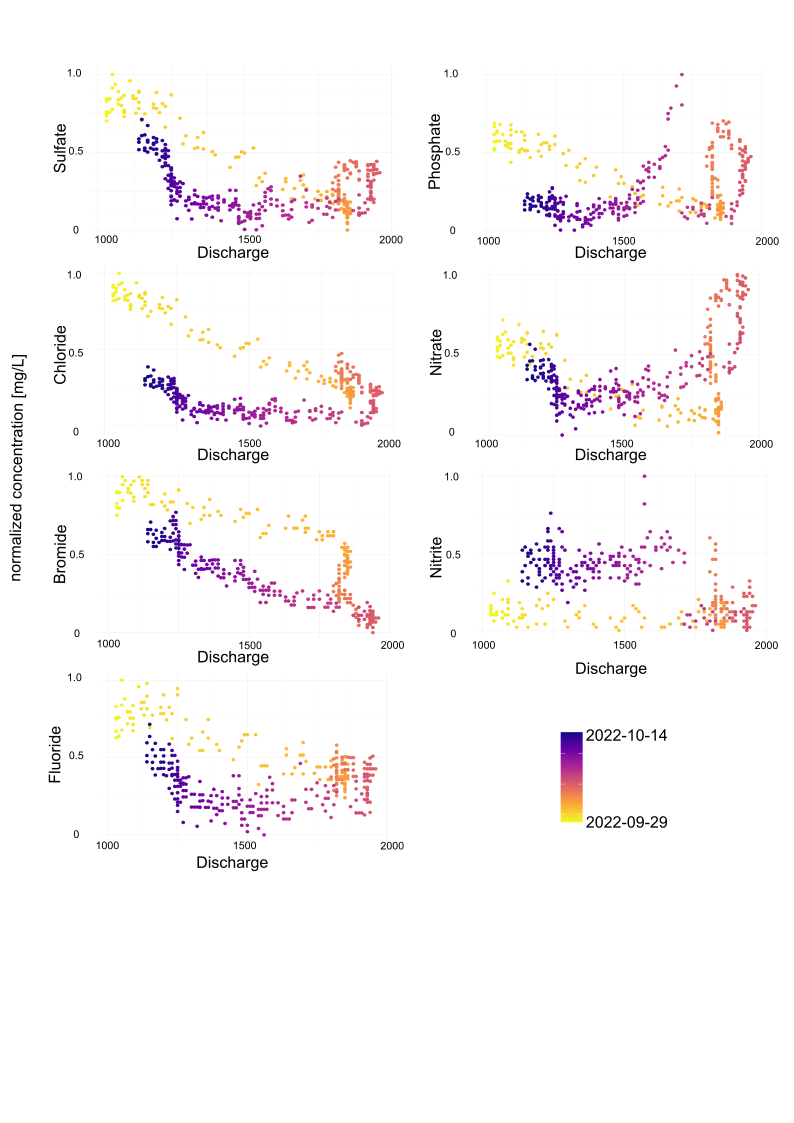


SI 9: Discharge and concentration during the flood event from 9^th^ January 2023 to 31^st^ January 2023.


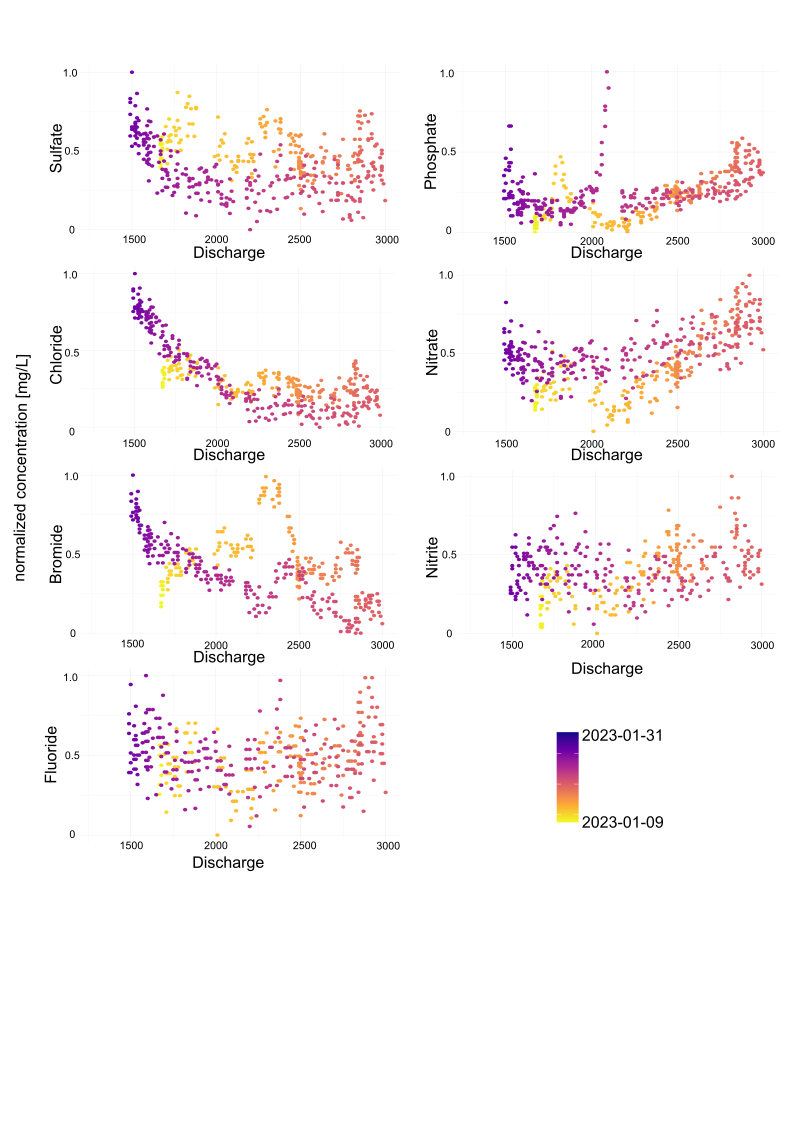


SI 10: This is a normalized version of Figure 2 from the main manuscript.


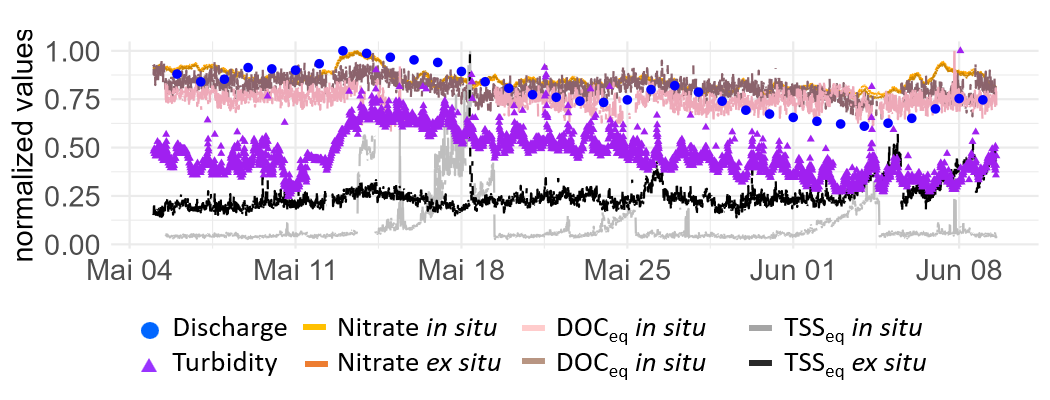

Supplement: Supplementary file 1 — (DOCX 871 KB) [file 10661_2025_14954_MOESM1_ESM.docx]
